# Fibroblast growth factor 23 is associated with carotid artery calcification in chronic kidney disease patients not undergoing dialysis: a cross-sectional study

**DOI:** 10.1186/1471-2369-14-22

**Published:** 2013-01-22

**Authors:** Masaru Nakayama, Yoshiki Kaizu, Masaharu Nagata, Yoriko Ura, Hirofumi Ikeda, Sho Shimamoto, Kazuyoshi Kuma

**Affiliations:** 1 Division of Nephrology and Clinical Research Institute, Department of Internal Medicine, National Kyushu Medical Center Hospital, 1-8-1 Jigyohama, Chuo-ku, Fukuoka 810-8563, Japan; 2 Department of Medicine and Clinical Science, Graduate School of Medical Science, Kyushu University, 3-1-1, Maidashi, Higashi-ku, Fukuoka, 812-8582, Japan

**Keywords:** Fibroblast growth factor 23, Vascular calcification, Carotid artery calcification, Chronic kidney disease

## Abstract

**Background:**

Fibroblast growth factor 23 (FGF23) is an important hormone in the regulation of phosphate metabolism. It is unclear whether FGF23 is associated with carotid artery calcification (CAAC) in predialysis patients. The present study aimed to clarify the relationship between FGF23 and CAAC in patients with chronic kidney disease (CKD) who were not on dialysis.

**Methods:**

One-hundred ninety-five predialysis CKD patients were enrolled in this cross-sectional study. CAAC was assessed using multidetector computed tomography, and the prevalence of CAAC was examined. Intact FGF23 was measured in each patient. The risk factors for CAAC were evaluated using a logistic regression model.

**Results:**

We found CAAC in 66% of the patients. The prevalence of CAAC significantly increased across CKD stages: it was 37% in CKD stages 1–2, 58% in stage 3; 75% in stage 4, and 77% in stage 5 (p < 0.01). In multivariate analysis, smoking, diabetes mellitus and log FGF23 were each identified as risk factors for CAAC. The study population was divided in quartiles of FGF23 levels. Compared with the lowest FGF23 quartile, each subsequent quartile had a progressively higher odds ratio (OR) for CAAC, adjusted for confounders (ORs [95% confidence interval] of 2.34 [0.78 to 7.31], 5.28 [1.56 to 19.5], and 13.6 [2.92 to 74.6] for the second, third, and fourth quartiles, respectively.

**Conclusions:**

The prevalence of CAAC is increased with the decline in the kidney function. FGF23 is independently related to CAAC in patients with CKD who are not on dialysis.

## Background

Vascular calcification is highly prevalent in patients with chronic kidney disease (CKD), including end-stage renal disease (ESRD) [1-3]. Arterial calcification in CKD patients occurs at the intimal as well as medial layers of the vasculature. Vascular intimal calcification, as well as vascular medial calcification, is associated with decreased survival in CKD [4]. We previously reported that there is a high prevalence of carotid artery calcification (CAAC) at the initiation of hemodialysis in ESRD patients [3] and that CAAC at the same time is an independent risk factor for cardiovascular events after the initiation of hemodialysis [5]. Therefore, the fact that cardiovascular disease is the most common cause of mortality in patients with CKD, including ESRD, appears to be due in part to the presence of excess vascular calcification.

Fibroblast growth factor 23 (FGF23) is a 251-amino-acid protein secreted by osteocytes in adults [6], and it is an important regulator that maintains serum phosphorus levels within the normal range in patients with CKD by increasing urinary phosphate excretion and decreasing dietary phosphorus absorption through the inhibition of 1,25-dihydroxyvitamin D (1,25(OH)_2_D) synthesis [7]. In patients with CKD, FGF23 levels are thought to increase as an appropriate compensatory mechanism to maintain a normal phosphate balance, in parallel with a declining capacity for renal phosphate excretion [8,9]. Notably, FGF23 is increased early in the course of kidney disease, well before the development of hyperphosphatemia and high FGF23 levels are thus considered to be among the earliest markers of disordered phosphorus metabolism in CKD [8,10]. The role of FGF23 in mortality and in CKD progression in patients with predialysis and in prevalent dialysis patients has been documented [11-15]; higher FGF23 levels are associated with higher mortality and a more rapid decline in kidney function.

The relationship between FGF23and vascular calcification is controversial. In subjects with normal kidney function, FGF23 is not associated with coronary artery calcification [16], while there is a positive association of FGF23with abdominal aortic calcification in older males [17]. Several studies have attempted to determine the relationship between FGF23 and vascular calcification in predialysis and prevalent dialysis patients, but the relationship is ambiguous. In hemodialysis patients, some studies have reported an independent positive correlation between FGF23 and peripheral and aortic calcification [18,19], but another study found that FGF23 was negatively correlated with peripheral calcification [20]. In predialysis CKD patients, FGF23 was not found to be associated with coronary artery calcification in a multivariate analysis [21]. A recent study found that serum FGF23 was associated with vascular calcification in patients at various CKD stages; however, 32% of the CKD patients examined were at stage 5D [22]. Based on these findings, it remains uncertain whether FGF23 is associated with vascular calcification in CKD patients who are not on dialysis. Therefore, the present study aimed to determine whether FGF23 levels are associated with CAAC in CKD patients who are not on dialysis.

## Methods

In this cross-sectional study, 195 patients with CKD who were not on dialysis, and who were admitted to our hospital for evaluation and education regarding their CKD, were enrolled. None of the patients had been administered active vitamin D_3_ or phosphate binders before admission. All of the patients provided written informed consent for the protocol, which was approved by the Ethics Committee of National Kyushu Medical Center Hospital.

After admission, all of the patients were provided a diet containing low salt and protein, and blood samples (for serum creatinine (SCr), C-reactive protein, hemoglobin, serum corrected calcium, serum phosphorus, intact parathyroid hormone [PTH], 1,25(OH)_2_D, and intact FGF23 levels) were obtained in the early morning after an overnight fast. Daily proteinuria was also measured. Fasting serum was stored at −80°C. Intact FGF23 levels were measured using an FGF23 ELISA kit (Kainos Laboratories Int, Tokyo, Japan). The primary and secondary incubations were performed at room temperature for 2 hours and 1 hour, respectively, and the chromogenic reaction was carried out at room temperature for 30 min. The assay for intact FGF23 has a lower limit of detection of 3 pg/mL and intraassay and interassay coefficients of variation (CV) of less than 10%. Intact PTH levels were measured by electrochemiluminescence immunoassay (ECLIA) (Elecsys PTH, Roche Diagnostics GmbH, Mannheim, Germany). The incubation was performed at 37°C for 18 min. The detection limit of the assay was 1.20 pg/mL. The intraassay CV was less than 2.8% and interassay CV was less than 3.4%. A radioimmunoassay was used to measure 1,25(OH)_2_D (Immunodiagnostic Systems Limited, Boldon, UK). The incubation was performed using the following three-step protocol: (1) incubation overnight at 2-8°C, (2) incubation at room temperature for 1 hour, and (3) incubation at room temperature for 30min. The lower detection limit was 2.1 pg/mL and the intra and interassay CVs were less than 15%. SCr, C-reactive protein, hemoglobin, serum calcium, serum phosphorus and proteinuria levels were measured by standard laboratory methods. The estimated glomerular filtration rate (eGFR) (mL/min/1.73m^2^) was calculated by using the Modification of the Diet in the Renal Disease equation for Japanese patients: 194 × SCr^-1.094^ × age^-0.287^ × 0.739 (if female) [23].

All of the enrolled patients were interviewed and underwent a clinical examination at presentation. Their medical history and outpatient records were also evaluated in detail. Demographic information (age and sex) and atherosclerotic risk factors (hypertension, history of smoking, dyslipidemia, and diabetes mellitus) at presentation were recorded for each patient. Cigarette smoking was evaluated as current or past. Body mass index was calculated as the weight in kilograms divided by height in meters squared.

**Figure 1:**
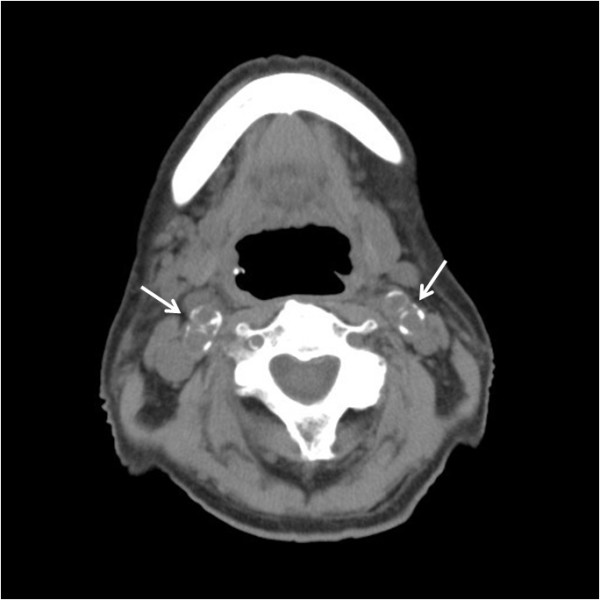
Carotid artery calcification detected by multi-detector computed tomography was seen at the origin of the bilateral internal and external carotid arteries (arrows).

Carotid computed tomography (CT) images were performed to detect calcification of common, internal or external carotid arteries, using a 16-row multidetector CT (MDCT) scanner (Multispeed Ultra 16x; GE Medical Systems, Milwaukee, WI) with 22.9 mm/s table speed, 512 × 512 pixel matrix, 0.625-mm slice thickness, 0.6-mm reconstruction index, 120 kV peak tube energy and autoregulation of tube currents (Figure 1). The mean radiation dose of CT scans was 3.8 ± 3.0 (SD) (mSV). Because there is currently no scoring system to quantify CAAC, unlike the Agatston scoring system for evaluating calcium mass of coronary arteries, the presence or absence of CAAC based on the visual assessment of CT scans was examined by a double-check system of two radiologists who were blinded to the clinical information. However, intra- and interrater agreement for the detection of CAAC were not assessed.

### Statistical analysis

Continuous data are expressed as either the means ± SD or the median (interquartile range) depending on their distribution, and categorical data are expressed as numbers (with %). Non-normally distributed variables (FGF23, intact PTH, and 1,25(OH)_2_D) were also log-transformed to approximately achieve a normal distribution and were then used in subsequent statistical analysis. The significance of differences between group with and that without CAAC was examined using the chi-square test for categorical data, the Wilcoxon rank sum test for nonparametric data, and the unpaired Student’s *t*-test for parametric data.

Demographic, traditional and non-traditional risk factors, serological data and the prevalence of CAAC were compared across quartiles of FGF23 or CKD stages using the chi-square test for categorical data, ANOVA for continuous variables that were approximately normally distributed, and the Kruskal-Wallis test for skewed continuous variables analysis. Dunnett’s test was then used after ANOVA and the Kruskal-Wallis test. A logistic regression model was applied to determine the traditional and nontraditional risk factors associated with CAAC. Covariates associated with CAAC which were significant in age- and sex-adjusted logistic regression analysis were selected as risk factors in the multivariate analysis. We also examined whether higher quartiles of FGF23 were associated with CAAC in a logistic regression analysis. Quartiles of FGF23 levels were chosen as the risk factors for CAAC, with the lowest quartile serving as the reference category. The interaction effect of FGF23 and other variables on the outcome was estimated by adding interaction terms between FGF23 categories assigned serial numerical codes and the status of other variables (below and above the median) to the relevant model. The odds ratio and the 95% confidence interval were calculated for each variable. Data were analyzed using the JMP9 statistical package (SAS Institute, Cary, NC, USA). A p value below 0.05 indicated a significant difference.

## Results

The mean age of the 195 patients (129 men and 66 women) in this study was 68.8 ± 11.3 years (range, 40–92 years). The primary causes of renal disease were chronic glomerulonephritis in 48 (24.6%) patients, diabetic nephropathy in 57 (29.2%), hypertensive nephrosclerosis in 60 (30.8%), other defined causes in 25 (12.8%), and unknown in 5 (2.6%). The clinical characteristics of the patients with and those without CAAC are summarized in Table 1. CAAC was found in 128 patients (66%). The mean age of the patients with CAAC was significantly higher than that of the patients without CAAC. The rates of hypertension, diabetes mellitus, smoking, and dyslipidemia in patients with CAAC were significantly higher compared with those in patients without CAAC. There were no significant differences in body mass index, proteinuria, C-reactive protein, and serum phosphate levels between the two groups. The values of hemoglobin, eGFR, and log 1,25(OH)_2_D in patients with CAAC were significantly lower than those in patients without CAAC, whereas PTH and FGF23 levels in patients with CAAC were significantly higher than those in patients without CAAC.

**Table 1:** Clinical characteristics of patients with and without carotid artery calcification

	**All (n = 195)**	**CAAC (+) (n = 128)**	**CAAC (−) (n = 67)**	**p values**
Age	68.8 ± 11.3	71.9 ± 9.4	62.7 ± 12.3	<0.01
Male	129 (66)	91 (71)	38 (57)	0.046
Hypertension	171 (88)	120 (94)	51 (76)	<0.01
Diabetes mellitus	92 (47)	72 (56)	20 (30)	<0.01
Smoking	110 (56)	82 (64)	28 (42)	<0.01
Dyslipidemia	146 (75)	102 (80)	44 (66)	0.03
Body mass index (kg/m^2^)	22.5 ± 4.5	22.3 ± 4.4	22.8 ± 4.6	0.45
Proteinuria (g/day)	1.2 (0.3-3.3)	1.8 (0.3-3.6)	0.9 (0.3-1.9)	0.06
C-reactive protein (mg/dL)	0.09 (0.04-0.22)	0.09 (0.04-0.24)	0.09 (0.03-0.19)	0.22
Hemoglobin (g/dL)	10.7 ± 2.1	10.3 ± 2.0	11.4 ± 2.2	<0.01
eGFR (ml/min/1.73m^2^)	23.2 (14.5-43.8)	20.0 (13.7-37.2)	34.3 (17.5-60.2)	<0.01
Serum corrected calcium (mg/dL)	9.4 ± 0.5	9.5 ± 0.6	9.3 ± 0.5	0.14
Serum phosphorus (mg/dL)	3.7 ± 0.7	3.8 ± 0.8	3.7 ± 0.7	0.80
Ca-P product (mg^2^/dL^2^)	35.3 ± 7.0	35.6 ± 7.2	34.6 ± 6.7	0.73
PTH (pg/mL)	73 (45–117)	82 (46–125)	56 (40–88)	0.03
1,25(OH)_2_Vitamin D (pg/mL)	25 (17–40)	24 (15–36)	30 (22–46)	<0.01
FGF23 (pg/mL)	64 (41–105)	73 (48–123)	50 (36–69)	<0.01

Values are expressed as the means ± SD, number (percent), or median (interquartile range).

*CAAC* carotid artery calcification, *eGFR* estimated glomerular filtration rate, *Ca-P* calcium-phosphorus, *PTH* parathyroid hormone, *FGF* fibroblast growth factor.

The clinical characteristics of the patients according to CKD stages are summarized in Table 2. Proteinuria in CKD stage 5 (n = 53) was significantly higher compared with that in CKD stages-1 and-2 (together, n = 27). There was no significant difference in C-reactive protein levels across CKD stages. Serum calcium levels were significantly lower and phosphate levels were significantly higher in CKD stage 5 patients compared with those in CKD stages 1–2 patients. The values of 1.25(OH)_2_D were significantly lower and PTH values were significantly higher in CKD stage 4 patients (n = 60) compared with those in CKD stages 1–2 patients. In contrast, significantly higher FGF23 levels in CKD stage 3 patients were already present compared with those in CKD stages 1–2 patients. In addition, the prevalence of CAAC was significantly increased across the CKD stages.

**Table 2:** Clinical characteristics of patients according to chronic kidney disease stages

	**CKD stage 1–2 (n = 27)**	**CKD stage 3 (n = 55)**	**CKD stage 4 (n = 60)**	**CKD stage 5 (n = 53)**	**P values**
Age	57.3 ± 12.9	68.3 ± 10.2*	72.3 ± 9.2*	71.1 ± 10.2*	<0.01
Male	11 (41)	41 (75)	42 (70)	35 (66)	0.02
Hypertension	15 (56)	47 (85)	58 (97)	51 (96)	<0.01
Diabetes mellitus	5 (19)	25 (45)	30 (50)	32 (60)	<0.01
Smoking	12 (44)	31 (56)	34 (57)	33 (62)	0.51
Dyslipidemia	16 (59)	40 (73)	48 (80)	42 (79)	0.19
Body mass index (kg/m^2^)	22.9 ± 3.3	23.3 ± 3.5	22.9 ± 5.1	21.0 ± 4.9	0.04
Proteinuria (g/day)	0.9 (0.4-1.8)	1.1 (0.1-3.4)	0.9 (0.1-3.5)	2.3 (0.7-4.0)**	0.03
C-reactive protein (mg/dL)	0.12 (0.05-0.23)	0.06 (0.03-0.17)	0.15 (0.04-0.26)	0.08 (0.05-0.20)	0.12
Hemoglobin (g/dL)	12.9 ± 1.9	11.6 ± 1.9*	10.3 ± 1.6*	9.0 ± 1.5*	<0.01
Serum corrected calcium (mg/dL)	9.6 ± 0.6	9.6 ± 0.6	9.4 ± 0.5	9.2 ± 0.5*	<0.01
Serum phosphorus (mg/dL)	3.4 ± 0.5	3.4 ± 0.5	3.6 ± 0.5	4.4 ± 0.9*	<0.01
Ca-P product (mg^2^/dL^2^)	32.6 ± 5.1	32.8 ± 5.0	34.1 ± 5.5	40.4 ± 8.5*	<0.01
Log PTH (pg/mL)	3.6 ± 0.4	3.9 ± 0.4	4.4 ± 0.6*	5.0 ± 0.6*	<0.01
Log 1,25(OH)_2_Vitamin D (pg/mL)	3.8 ± 0.4	3.6 ± 0.4	3.2 ± 0.4*	2.6 ± 0.6*	<0.01
Log FGF23 (pg/mL)	3.5 ± 0.5	3.9 ± 0.4**	4.3 ± 0.7*	5.0 ± 0.9*	<0.01
CAAC	10 (37)	32 (58)	45 (75)	41 (77)	<0.01

Values are expressed as the means ± SD, number (percent), or median (interquartile range).

*CKD* chronic kidney disease, *Ca-P* calcium-phosphorus, *PTH* parathyroid hormone, *FGF* fibroblast growth factor, *CAAC* carotid artery calcification.

*p < 0.01, **p < 0.05 vs. CKD stage 1–2.

Table 3 shows the results of the logistic regression analysis for having CAAC. Adjusted for age and sex, hypertension, diabetes mellitus, smoking, proteinuria, calcium-phosphorus product, and log FGF23 were significantly associated with CAAC. Multivariate analysis showed that diabetes mellitus, smoking, and log FGF23 remained as independent determinants for CAAC.

**Table 3:** Odds ratios for carotid artery calcification in patients with chronic kidney disease

	**Age- and sex- adjusted**			**Multivariate adjusted**^**a)**^		
**Variables**	**OR**	**95% CI**	**p values**	**OR**	**95% CI**	**p values**
Hypertension	3.70	1.35-10.6	<0.01	1.63	0.50-5.35	0.41
Diabetes mellitus	3.62	1.81-7.53	<0.01	2.27	1.01-5.27	0.048
Smoking	3.26	1.57-6.97	<0.01	2.39	1.14-5.12	0.02
Dyslipidemia	1.95	0.93-4.12	0.08			
Body mass index (kg/m^2^)	1.02	0.94-1.09	0.65			
Proteinuira (g/day)	1.22	1.06-1.43	<0.01	1.09	0.92-1.30	0.32
C-reactive protein (mg/dL)	1.58	0.62-5.69	0.37			
Hemoglobin (g/dL)	0.86	0.73-1.01	0.06			
Serum phosphorus (mg/dL)	1.34	0.86-2.15	0.21			
Ca-P product (mg^2^/dL^2^)	1.06	1.00-1.12	0.03	0.98	0.92-1.30	0.62
eGFR (ml/min/1.73m^2^)	0.99	0.97-1.00	0.09			
Log FGF23 (1SD increase)	1.79	1.12-2.87	<0.01	1.75	1.01-3.04	0.01

^a)^ Adjusted for age and covariates which were significant in an age- and sex-adjusted analysis.

*OR* odds ratio, *CI* confidence interval, *Ca-P* calcium-phosphorus, *eGFR* estimated glomerular filtration rate, *FGF* fibroblast growth factor.

The study population was divided into quartiles of FGF23 levels (Table 4). With increasing quartiles of FGF23, mean serum phosphate and calcium-phosphorus product levels were higher, and mean hemoglobin and eGFR were lower. In addition, the prevalence of CAAC significantly increased with ascending quartiles of FGF23. Table 5 shows the odds ratios for having CAAC across quartiles of FGF23 levels obtained with the logistic regression model. In a crude model, both the third and fourth FGF23 quartiles were associated with CAAC. After being adjusted for demographic, traditional, and nontraditional risk factors (model^4^), the third and fourth FGF23 quartiles remained independently associated with CAAC. In addition, the effect of FGF23 and that of other variables, such as proteinuria, serum phosphorus, serum calcium, intact PTH, and eGFR, on the risk for CAAC were independent of each other (p for interaction; 0.61, 0.46, 0.11, 0.69, and 0.50, respectively).

**Table 4:** Clinical characteristics of the CKD patients according to quartiles of FGF23 levels

	**FGF23-Quartile 1**	**FGF23-Quartile 2**	**FGF23-Quartile 3**	**FGF23-Quartile 4**	
	**(n = 47; FGF23, 11–40)**	**(n = 50; FGF23, 41–63)**	**(n = 49; FGF23, 64–103)**	**(n = 49; FG23, 105–2010)**	**p values**
Age (years)	67.7 ± 14.2	68.5 ± 10.6	68.2 ± 10.3	70.5 ± 10.0	0.65
Male	26 (55)	33 (66)	35 (71)	35 (71)	0.31
Hypertension	38 (81)	37 (74)	48 (98)	48 (98)	<0.01
Diabetes mellitus	20 (43)	20 (40)	29 (59)	23 (47)	0.23
Smoking	23 (49)	29 (58)	30 (61)	28 (57)	0.66
Dyslipidemia	35 (74)	33 (66)	35 (71)	43 (88)	0.06
Body mass index (kg/m^2^)	22.2 ± 2.5	22.4 ± 5.7	22.7 ± 5.4	22.7 ± 3.5	0.95
Proteinuria (g/day)	1.2 (0.1-3.3)	0.7 (0.1-2.6)	2.2 (0.4-4.0)	1.2 (0.4-3.6)	0.14
C-reactive protein (mg/dL)	0.05 (0.02-0.12)	0.09 (0.03-0.22)	0.13 (0.05-0.26)	0.09 (0.05-0.35)*	0.01
Hemoglobin (g/dL)	11.5 ± 2.2	11.1 ± 2.1	10.6 ± 2.0	9.6 ± 1.7**	<0.01
Serum corrected calcium (mg/dL)	9.5 ± 0.6	9.4 ± 0.5	9.5 ± 0.5	9.3 ± 0.6	0.19
Serum phosphorus (mg/dL)	3.4 ± 0.5	3.5 ± 0.6	3.6 ± 0.5	4.4 ± 0.9**	<0.01
Ca-P product (mg^2^/dL^2^)	32.5 ± 5.6	33.3 ± 6.0	34.4 ± 4.2	40.7 ± 8.5**	<0.01
eGFR (ml/min/1.73m^2^)	46.9 (27.0-68.9)	33.6 (17.3-57.9)	22.0 (16.0-35.0)**	12.7 (9.0-17.2)**	<0.01
CAAC	23 (49)	29 (58)	37 (76)	39 (80)	<0.01

Values are expressed as the means ± SD, number (percent), or median (interquartile range).

*CKD* chronic kidney disease, *FGF* fibroblast growth factor, *Ca-P* calcium-phosphorus, *eGFR* estimated glomerular filtration rate, *CAAC* carotid artery calcification.

*p < 0.05, **p < 0.01 vs. FGF23-Quartile 1.

**Table 5:** Odds ratios for carotid artery calcification according to quartiles of FGF23 levels

		**OR**	**95% CI**	**p values**
Model^1^	FGF23-quartile 1	ref.		
	FGF23-quartile 2	1.44	0.65-3.24	0.37
	FGF23-quartile 3	3.22	1.38-7.85	<0.01
	FGF23-quartile 4	4.07	1.69-10.4	<0.01
Model^2^	FGF23-quartile 1	ref.		
	FGF23-quartile 2	1.44	0.59-3.59	0.42
	FGF23-quartile 3	3.62	1.40-9.89	<0.01
	FGF23-quartile 4	4.05	1.54-11.3	<0.01
Model^3^	FGF23-quartile 1	ref.		
	FGF23-quartile 2	1.85	0.67-5.23	0.23
	FGF23-quartile 3	3.59	1.28-10.7	0.01
	FGF23-quartile 4	4.31	1.52-13.0	<0.01
Model^4^	FGF23-quartile 1	ref.		
	FGF23-quartile 2	2.34	0.78-7.31	0.13
	FGF23-quartile 3	5.28	1.56-19.5	<0.01
	FGF23-quartile 4	13.6	2.92-74.6	<0.01

*FGF* fibroblast growth factor, *OR* odds ratio, *CI* confidence interval.

Model^1^, crude.

Model^2^, adjusted for age and sex.

Model^3^, adjusted for model^2^ plus hypertension, diabetes mellitus, smoking, and dyslipidemia.

Model^4^, adjusted for model^3^ plus eGFR, serum phosphorus, serum calcium, intact PTH, and proteinuria.

*eGFR* estimated glomerular filtration rate, *PTH* parathyroid hormone.

## Discussion

The principal finding in the present study was that FGF23 was independently associated with CAAC in CKD patients who were not on dialysis. The prevalence of CAAC was significantly increased as the kidney function declined. FGF23levels were significantly higher in CKD stage 3 patients than in CKD stages 1–2 patients. In contrast, serum phosphate levels were significantly higher only in CKD stage 5patients compared with CKD stages 1–2 patients, and were not related to CAAC. These results suggest that measurement of FGF23 is more important than measurement of serum phosphorus for the detection of risk factors associated with CAAC.

In ESRD patients, hyperphosphatemia is thought to be associated with vascular calcification [24-26]. In contrast, the association of serum phosphorus levels with vascular calcification in predialysis CKD patients has remained uncertain. Some studies have shown no significant correlation between serum phosphorus and vascular calcification [1,27], while it has been demonstrated that higher serum phosphate levels, although still within the normal range, are associated with a greater prevalence of vascular and valvular calcification in subjects with moderate CKD [28]. Most patients develop hyperphosphatemia only at CKD stages 4 and 5 [29,30], despite the progressive elevation of serum PTH and FGF23 levels as a defense mechanism to prevent an increase in serum phosphate levels. The present study also showed that serum phosphate levels were significantly high only in CKD stage 5 patients compared with CKD stages 1–2 patients, whereas significantly higher FGF23 levels were already observed in CKD stage 3 patients compared with CKD stages 1–2 patients. These findings are in agreement with previous studies [8,10] reporting that an increase in FGF23 levels precedes an increase in serum phosphate levels in CKD patients, and they may support the hypothesis that elevated FGF23 concentrations are an early abnormality of disordered phosphorus metabolism in CKD patients [6].

Vascular calcification appears early in the course of CKD (i.e., well before ESRD is reached) [31]. One study reported that a high prevalence (64%) of coronary artery calcification was found in CKD patients with a median creatinine clearance of 37 mL/min/1.73m^2^ and a median serum phosphorus of 3.8 mg/dL [32]. In the present study, a high prevalence of CAAC was already present in CKD stage 3 patients, who also had a mean serum phosphate level of 3.4 mg/dL. Moreover, concerning the recent findings that high normal serum phosphate levels are associated with vascular calcification in predialysis CKD patients [28], it is possible that even lower serum phosphate levels contribute to vascular calcification. In addition, given the present finding that FGF23 levels, but not serum phosphate levels, were identified as an independent factor for CAAC, FGF23 measurement appears to be more important for detecting CAAC-associated risk factors in CKD patients who are not undergoing dialysis.

Although there is the possibility that elevated FGF23 levels affect bone metabolism or vascular calcification directly and/or indirectly, the precise mechanism of such an effect remains unknown. The potential finding of FGF23 levels as a biomarker of CAAC in the present study cannot lead to the conclusion of a real role of FGF23 in the pathogenesis of vascular calcification.

The main physiological stimuli of FGF23 secretion are increased dietary phosphorus intake and increased 1,25(OH)_2_D levels [33]. Accordingly, in the present study, we studied patients who had not been administered active vitamin D_3_ or phosphate binders, to more precisely determine whether FGF23 levels are associated with vascular calcification. Burnett et al. demonstrated that dietary phosphate loading increases the fractional excretion of phosphate and circulating FGF23 in 66 healthy males and females, suggesting that dietary phosphate is an important regulator of circulating FGF23 levels in humans [34]. However, an association of phosphate loading with changes in FGF23 levels has not been fully documented in CKD patients. In a small, randomized controlled trial of CKD patients, Isakova et al. recently reported that CKD patients receiving a higher phosphorus diet plus placebo for 2 weeks demonstrated a significant increase in FGF23 levels, with no significant changes over time in serum phosphate levels, suggesting an important effect of dietary phosphorus intake on FGF23 levels rather than serum phosphate levels [35]. However, it remains unknown whether high dietary phosphate loading contributes to the development of vascular calcification in CKD patients.

Moderately uremic mice fed a high phosphate diet are not hyperphosphatemic but have a significant rise in serum FGF23 levels that are significantly correlated with arterial medial calcification [36]. In the present study, phosphate intake or loading was not assessed in a quantitative manner over prolonged time periods before the patients’ admission. Accordingly, it remains unknown whether elevated FGF23 levels in some of our study population might reflect a reaction to phosphate loading, and the effect of phosphate loading on vascular calcification could not be assessed.

A previous study demonstrated that in normophosphatemic CKD patients there was a significant decrease in FGF23 levels, but not serum phosphorus levels, in patients treated with the non-calcium containing phosphate binder, sevelamer, for 6 weeks, whereas there was no decrease in FGF23 in calcium acetate-treated patients [37]. However, it is unclear whether a decrease in FGF23 by non-calcium containing phosphate binders is related to a simultaneous decrease in vascular calcification in CKD patients not on dialysis. Therefore, long, randomized-intervention studies are required to clarify the effect of non-calcium containing phosphate binders on changes in both FGF23 and vascular calcification in CKD.

The current study has some limitations. First, the patient cohort was relatively small. Second, the multidetector CT assessment depended on the presence or absence of CAAC, but calcium content was not scored. If the CAAC score is quantified, then the associated risk factors may be more precisely documented, and the relationship between CAAC scores and FGF23 levels should be defined. Third, serum klotho levels were not examined in the present study. It has been reported that klotho deficiency contributes to soft-tissue calcification in CKD [38]. FGF23 generally requires its cofactor, klotho, for its activity. High levels of FGF23, as well as deficient renal klotho expression and function, are found in CKD patients. It is possible that, in the absence of klotho, increased FGF23 levels exert pro-calcific effects via non-specific low affinity binding to its receptors [39-41]. A recent study showed that in human arteries, restoration of klotho expression by vitamin D receptor activators could unmask anticalcific effect of FGF23 [42]. Further investigations are warranted to determine whether high FGF23 levels, independent of klotho deficiency, contribute to the development of vascular calcification in CKD patients.

## Conclusions

In CKD patients, the prevalence of CAAC is significantly increased across the CKD stages. Our findings also demonstrate that FGF23 is independently associated with CAAC. In the future, additional investigations are warranted to further clarify the precise mechanisms by which FGF23 contributes to the development of vascular calcification.

## Competing interests

The authors declare that they have no competing interests.

## Authors’ contributions

MN^1^ was involved in the study design, sample collection, analysis and interpretation of the data and in the writing of the report. YK acquired the data and participated in the interpretation of the data. MN^2^ participated in the analysis and interpretation of the data. YU, HI, SS and KK participated in the sample collection. All authors have read and approved the final version of the manuscript.

## Pre-publication history

The pre-publication history for this paper can be accessed here:

http://www.biomedcentral.com/1471-2369/14/22/prepub
